# Protecting whose welfare? A document analysis of competition regulatory decisions in four jurisdictions across three harmful consumer product industries

**DOI:** 10.1186/s12992-024-01076-2

**Published:** 2024-10-02

**Authors:** Benjamin Wood, Chrissa Karouzakis, Katherine Sievert, Sven Gallasch, Gary Sacks

**Affiliations:** 1 Global Centre for Preventive Health and Nutrition, Institute for Health Transformation, School of Health and Social Development, Deakin University, 221 Burwood Highway, Burwood, Victoria 3125 Australia; 2 Deakin Law School, Deakin University, Burwood, 221 Burwood Highway, Victoria 3125 Australia

**Keywords:** Industrial epidemics, Commercial determinants of health, Competition policy, Market power, Consumer welfare, Health in all policies

## Abstract

**Background and methods:**

Competition regulation has a strong influence on the relative market power of firms. As such, competition regulation can complement industry-specific measures designed to address harms associated with excessive market power in harmful consumer product industries. This study aimed to examine, through a public health lens, assessments and decisions made by competition authorities in four jurisdictions (Australia, South Africa, the United States (US), and the European Union (EU)) involving three harmful consumer product industries (alcoholic beverages, soft drinks, tobacco). We analysed legal case documents, sourced from online public registers and dating back as far as the online records extended, using a narrative approach. Regulatory decisions and harms described by the authorities were inductively coded, focusing on the affected group(s) (e.g., consumers) and the nature of the harms (e.g., price increases) identified.

**Results:**

We identified 359 cases published by competition authorities in Australia (*n* = 202), South Africa (*n* = 44), the US (*n* = 27), and the EU (*n* = 86). Most cases (*n* = 239) related to mergers and acquisitions (M&As). Competition authorities in Australia, the US, and the EU were found to make many decisions oriented towards increasing the affordability and accessibility of alcohol beverages, soft drinks, and tobacco products. Such decisions were very often made despite the presence of consumption-reduction public health policies. In comparison, South Africa’s competition authorities routinely considered broader issues, including ‘Black Economic Empowerment’ and potential harms to workers.

**Conclusion:**

Many of the competition regulatory decisions assessed likely facilitated the concentration of market power in the industries we explored. Nevertheless, there appears to be potential for competition regulatory frameworks to play a more prominent role in promoting and protecting the public’s health through tighter regulation of excessive market power in harmful consumer product industries.

## Introduction

Harmful consumer product industries are a major driver of the substantial and increasing global burden of preventable death and disease [1–6]. It has been estimated that the products and practices of just three industries – tobacco, alcohol, and ultra-processed food and drink – currently contribute to more than one quarter of all preventable deaths worldwide [3].

Thus far, policy efforts to address the health-related harms associated with harmful consumer product industries have mostly focused on industry-specific measures [7]. Some industry-specific measures have made important contributions to improving population health outcomes. These include measures such as comprehensive bans on tobacco advertising and sponsorship [8], minimum unit pricing of alcohol [9], and taxes on sugary drinks [10]. Nevertheless, policy efforts to curb the rising global burden of preventable death and disease propagated by harmful consumer product industries have been largely piecemeal and inadequate [1, 4, 7, 11]. In this respect, it is helpful to recognise that population health is not just an outcome of public health policies or health sector programs, but that it is also shaped to a large degree by government policy and regulatory frameworks beyond the health sector [11, 12].

In this paper, we contend that competition regulation is a pertinent example of a regulatory framework that likely has a considerable influence on population health outcomes, particularly via its influence on various upstream determinants of health. In many jurisdictions, competition regulation, which typically includes the regulation of mergers, cartels, and abuses of market power, has a strong influence on the relative market power of firms [13, 14]. Accordingly, competition regulation represents a potentially important tool to address harms associated with the presence and misuse of market power, including in harmful consumer product industries. In this paper, we broadly refer to market power as the power of a firm to shape prices, innovation pathways, industry standards, and the overall ‘architecture of the market’ to suit its own interests [15]. Moreover, in many contexts, market power very often readily translates into the political power to influence government regulation, policy, and public opinion [16–20].

A number of scholars have raised concerns about how, at least in recent decades, competition regulation has largely facilitated rising market power across many parts of the global economy [14, 21–23]. One commonly proposed explanation behind such concerns is that the goal of competition policy in many jurisdictions has been increasingly shaped by a neoliberal discourse that has shifted the focus of competition regulation onto a narrow set of economic concerns [15, 24–26]. Indeed, to inform their decision-making, many competition authorities reportedly use some form of the so-called ‘consumer welfare’ standard, which ostensibly places the focus of competition regulation onto lowering consumer prices and increasing output [27]. Prices and output can be important considerations in competition analysis. Nevertheless, in their analysis of ‘consumer welfare’, which is often conducted in accordance with neoclassical economic theory, competition authorities often remain indifferent to the nature of the product or service in question [25]. In the case of harmful consumer product industries, such indifference has the potential to foster regulatory decisions that are inconsistent with public policies seeking to reduce the use or consumption of the same commodity in question [28]. As an illustrative example, competition regulatory decisions designed to lower the price and maximise the production of alcoholic products are likely to run counter to ‘best practice’ public health policies, such as excise taxes and marketing restrictions, designed to reduce harms associated with the consumption of alcoholic products [29].

With a few exceptions [25, 28], minimal attention has been devoted to analysing the ways in which competition regulation in harmful consumer product industries has potentially undermined or promoted public health policy and, more broadly, the public’s health. With this in mind, this study aimed to examine, through a public health lens, assessments and decisions made by various competition authorities with respect to a selection of key harmful consumer product industries. Findings were used to inform discussion on opportunities to strengthen synergies between competition regulation and public health policies targeting these industries. The purpose of this study was to support broader research and advocacy efforts seeking to address the commercial determinants of ill-health and inequity.

## Methods

In this study, we conducted a document analysis of assessments and decisions made by competition authorities in four jurisdictions (Australia, South Africa, the United States, and the European Union) with respect to cases involving three harmful commodity industries (alcoholic beverages, soft drinks, tobacco) through a public health lens. Further details are provided below.

### Study sample

We analysed competition-related cases involving competition authorities in Australia (the Australian Competition and Consumer Commission (ACCC)), South Africa (the Competition Tribunal of South Africa (CTSA)), the United States (the United States Department of Justice (DOJ) and Federal Trade Commission (FTC)), and the European Union (the European Commission (EC)). We included the United States (US) and European Union (EU) in our sample because these are widely considered the two most dominant competition policy regimes in the world, and also because many major corporations have their headquarters in these jurisdictions [30]. Australia was included because the authors were based in an Australian university, and this jurisdiction is the main focus of their work. South Africa was selected because it is often portrayed as an exemplar of how to integrate public interest considerations into competition regulation [31–33].

To manage scope, the study was limited to three harmful commodity industries: alcoholic beverages, soft drinks, and tobacco. These three industries were chosen because, as noted in the previous section, they are major contributors to the large and rising global burden of preventable death and disease. We opted to focus on the soft drink industry (a subset of the UPF industry) instead of the entire UPF industry largely to avoid issues relating to market definitions in competition assessments [34]. For example, while branded breakfast cereal manufacturers and soft drink manufacturers would generally be considered as part of the UPF industry, these products would typically not be considered as part of the same product market by competition authorities. In competition cases in which market definition serves as a key analytical tool, such as merger control, UPF manufacturers in different product markets may not be seen as being in competition (at least from the demand side) [34].

## Data collection and analysis

Data were collected from the online public registers curated by the ACCC [35], the CTSA [36], the Antitrust Division of the US DOJ [37], the US FTC [38], and the EC [39]. We chose to examine documents published by the CTSA and not the Competition Commission of South Africa because, at the time of data extraction, the latter did not appear to have an archive containing published case documents on its website. The search was conducted between April and May 2023. Cases pertaining to the three selected industries were found by using the search tools and industry filters specific to the website in question. Within each online case folder, documents including competition-related assessments and decisions were identified. Documents that were not published in English were excluded from the analysis. Case folders without a document containing a competition-related assessment (e.g., those related to consumer protection cases) were also excluded from the analysis. Additionally, we chose to exclude ‘state aid’ cases, which are specific to the EU, from the analysis to maintain case comparability across jurisdictions. No restrictions were placed on the year of publication.

Following the search and screening phase, documents were retrieved and tabulated by one of the authors (CK) in Excel. From the included documents, text relating to the following characteristics or subjects were extracted: (i) competition authority and jurisdiction in question; (ii) type of case (e.g., merger and acquisition, price-fixing cartel behaviour, exclusive dealing arrangements); (iii) names of party or parties involved and their primary industry or industries; iii) year the case was published; (iv) scale and nature of any harms described or proposed by the authority in question that may have been caused, or that could potentially be caused, by the conduct in question (e.g., an increase in consumer price, a loss of employment); and (v) any corresponding regulatory decisions made (e.g., the unconditional approval of a merger; the prohibition of price-fixing cartel behaviour). Proposed or described harms described by the authorities were inductively coded using the software program NVivo, focusing on the affected group(s) (e.g., consumers) and its nature (e.g., price increase). Regulatory decisions were also inductively coded using the same program. Collaborative coding of random samples of data (approximately 25% of data) was conducted between two authors (CK and BW), with the remaining data sources coded by one researcher (CK). Codes were discussed and finalised during a series of meetings among the author group. Data were synthesised using a narrative approach.

## Results

We identified 359 cases with publicly accessible documents pertaining to assessments and decisions made by competition authorities in Australia (*n* = 202), South Africa (*n* = 44), the United States (*n* = 27), and the European Union (*n* = 86). Australia likely accounted for the majority of cases identified in this study because, in contrast to the other authorities, the ACCC assessed a large number of merger and acquisition (M&A) cases involving local alcohol retail outlets, as well as promotion deals labelled as exclusive dealing arrangements. Further details are provided below.

Overall, 251 cases directly related to the alcoholic beverage industry, 69 cases to the soft drink industry, and 39 cases to the tobacco industry (Table 1). With respect to the type of cases, 239 related to mergers and acquisitions (M&As), 92 related to unilateral actions (e.g., exclusive dealing arrangements, abuse of a dominant position), and 28 related to coordinated actions (e.g., price and/or supply coordination among rivals, collective bargaining arrangements) (Table 2).

**Table 1:** Number of cases involving the alcoholic beverage, soft drinks, and tobacco industries examined in the study, disaggregated by the competition authority and industry in question

Authority (Jurisdiction)	Number of cases with documents analysed			Total
	Alcoholic beverages	Soft drinks	Tobacco	
Australian Competition and Consumer Commission (Australia)	161	28	13	202
Competition Tribunal of South Africa (South Africa)	27	9	8	44
Department of Justice and the Federal Trade Commission (United States)	17	3	7	27
European Commission (European Union)	46	29	11	86
**Total**	251	69	39	359

**Table 2:** Number of cases involving the alcoholic beverage, soft drinks, and tobacco industries examined in the study, disaggregated by the competition authority and type of case in question

Authority (Jurisdiction)	Number of cases with documents analysed			Total
	Mergers & acquisitions	Unilateral actions	Coordinated actions	
Australian Competition and Consumer Commission (Australia)	109	80	13	202
Competition Tribunal of South Africa (South Africa)	41	3	0	44
Department of Justice and the Federal Trade Commission (United States)	17	0	10	27
European Commission (European Union)	72	9	5	86
**Total**	239	92	28	359

### Australian Competition and Consumer Commission

We found 202 cases with documents published by the ACCC, dating back to 1999. Regarding the types of cases examined, M&As (*n* = 109) accounted for the majority. Many of these were M&As proposed by Australian supermarket company Woolworths involving the acquisition of alcohol retailing operations (*n* = 56). The ACCC did not raise any competition-related concerns in most of the M&A cases analysed in this study (*n* = 97). Accordingly, the authority approved many M&As without conditions.

Regarding the M&A cases in which the ACCC raised competition-related concerns, we found that the authority opted to oppose two, while its preliminary opposition to two other acquisitions likely contributed to these being withdrawn by the parties in question. In these four cases [40–43], we did not find explicit mention of the actor group, such as consumers or workers, at risk of being harmed by the conduct in question. In three of these cases, however, all of which involved Woolworths proposing to acquire an alcohol retailer [40, 41, 43], we found that the ACCC alluded to ways in which the loss of competition, including as a result of increased market concentration and barriers to market entry, could potentially harm consumers in the form of increased prices and reduced consumer choice. As an example, in one case, the ACCC expressed concern that the acquisition would likely result in a substantial lessening of competition, before commenting that competition was important to encourage, inter alia, price competition, specials and promotions, and increased consumer choice [43].

In seven M&A cases, the ACCC opted to conditionally approve the transaction after accepting the legal undertakings proposed by the parties to alleviate concerns raised by the authority in its assessment. In these cases, the most common concern raised by ACCC related to how the transaction could potentially harm consumers, including in the form of increased prices and reduced frequency of promotions. As an illustrative example, in its assessment of a proposed acquisition by Asahi Group Holdings of Carlton & United Breweries (CUB), the ACCC concluded that ‘the removal of competitive rivalry between Asahi and CUB would be likely to enable a combined CUB and Asahi to profitably increase the price of cider products by a significant margin’ [44]. To address such concerns, the ACCC accepted a remedy whereby Asahi agreed to divest part of its cider and beer operations in Australia. In another M&A case involving tobacco companies Scandinavian Tobacco Group and Swedish Match, the ACCC considered that the divestiture of various assets would be sufficient to address its concerns, including that the proposed acquisition would potentially result in ‘the removal of pricing constraints on the merger parties leading to increased wholesale prices’ and a ‘reduction in the frequency of discounting or promotional offers’ in the Australian cigar market [45].

In our analysis, exclusive dealing arrangements (*n* = 79) were the second most frequent type of competition case assessed by the ACCC. Most of these related to promotional deals involving the tying or bundling of free or discounted alcoholic or soft drink products to a particular service (e.g., subscription to an online news service [46]). In most of these cases, the ACCC did not raise any competition-related concerns. In some cases, the ACCC contended that such actions could potentially provide ‘public benefits’ in the form of free or discounted goods, as well as by pressuring competitors to offer similar promotions.

We found seven cases involving price or supply coordination between competitors. In three of these cases, we identified that the ACCC explicitly considered public health concerns in its decision-making. As an example, in 2017, the ACCC opted to prohibit an arrangement proposed by British American Tobacco Australia, Imperial Tobacco Australia, and Philip Morris, in which the parties sought permission to take coordinated action against retailers and wholesalers supplying illicit tobacco [47]. In its assessment, the ACCC expressed concern that, inter alia, the arrangement would likely ‘result in detriment by undermining public health outcomes and enforcement agencies’ efforts to enforce tobacco control laws and their underlying policies’ [47]. The ACCC further argued that the arrangements would likely give the companies ‘a quasi-regulatory role in circumstances where their commercial incentives do not entirely align with those of government’, and that this ‘may be inconsistent with World Health Organization guidelines for the implementation of Australia’s obligations under international agreements about tobacco control’ [47].

In two other related cases, the ACCC approved – and subsequently re-approved – a proposal by the State of Queensland’s Office of Liquor and Gaming Regulation (OLGR) to organise price controls (e.g., a requirement to offer serves of lower alcohol beverages at lower prices than full strength beverages) and supply controls (e.g., ban on drinking games) among licensed premises in various local areas [48, 49]. Although the ACCC considered that the price and supply controls would likely result in some ‘public detriment through potential effects on price and consumer choice’, this was reportedly outweighed by the public benefit in the form of community-based alcohol-related harm minimisation [48].

### Competition Tribunal of South Africa

We found 44 cases with documents published by the CTSA, dating back to 2000. The majority (*n* = 41) of these cases related to M&As. We found that competition-related concerns were raised on four occasions. In these cases, we did not find explicit mention of the actor group likely to be harmed by the competition-related aspects of the conduct in question. Instead, the focus appeared to be on structural issues. For instance, in two of these cases, the raised competition-related concerns largely centred on how the transaction in question would likely result in dominant market shares and high levels of concentration in a market with considerable barriers to entry [50, 51]. We found that the CTSA subsequently accepted the divestiture of several alcohol retail outlets as a remedy for one of these cases [50]. We were unable to identify the prescribed remedies for the other case. In another M&A case, the main competition-related concern appeared to centre on how the merging party would likely be able to leverage its dominance to exclude competing companies from accessing various distribution channels [52]. In this instance, the authority accepted a set of behavioural conditions preventing the merging parties from engaging in exclusionary forms of conduct.

In accordance with their mandate, we also found that South Africa’s competition authorities expressed public interest-related concerns in seven M&A cases. Specifically, concerns relating to employment numbers were raised on six occasions. As an illustrative example, in one M&A case involving South African Breweries and Diageo South Africa, South Africa’s Competition Commission raised concerns that the transaction would likely result in retrenchments of employees in a specific plant [53]. To address these concerns, the merging parties agreed to re-deploy the relevant staff within the broader corporate group. On three occasions, concerns related to the share-ownership by black persons – as part of the South African policy commonly referred to as ‘Black Economic Empowerment’ – were voiced by the authorities. As a remedy to one proposed transaction, the CTSA required Coca-Cola Beverages Africa to increase its current share ownership by qualifying ‘Black Economic Empowerment’ groups from 11 to 20%, and to sell 20% of the equity of one its subsidiaries to a qualifying black company or consortium [54].

Furthermore, concerns relating to the competitiveness of domestic industry and small-to-medium sized enterprises were raised in two cases. In the above case involving Coca-Cola Beverages Africa, the merging party was also required to: (i) invest 400 million South African Rand (approximately 22 million USD at the time of writing) into developing the ‘distribution and retail aspects’ of the company’s South African soft drink business; (ii) provide skills training to 25,000 black retailers of the company’s products; (iii) provide small local companies with 10% space in the coolers and fridges supplied by the company; and (iv) establish a fund for business development in the agriculture value chain, including for ‘historically disadvantaged developing farmers’ [54]. As another example, in one M&A case involving Anheuser-Busch InBev and SABMiller, the merging parties were required to invest 610 million South African Rand (approximately 34 million USD at the time of writing) into ‘agricultural development’ in accordance with the South African government’s goal of increasing the capacity of domestic hop and barley producers to meet ‘local demand’ [55].

### United States Department of Justice and Federal Trade Commission

We found 27 cases with documents published by the US DOJ (*n* = 20) and FTC (*n* = 7), dating back to 1965. Of these cases, 17 related to M&As. Three mergers scrutinised by the US competition authorities were blocked by US courts. Two of these blocked mergers took place before 1972, and in both instances, the main concerns centred on market dominance and concentration. In 1970, for instance, a District Court opposed tobacco company R.J. Reynolds’ proposed merger with a number of shipping companies arguing that, inter alia, the merging party would likely ‘enhance and entrench’ its dominance to the detriment of competition [56]. In 1971, another District Court effectively ordered beer company Pabst Brewing to undo its acquisition of a rival beer company undertaken several years earlier [57], citing an earlier judgement made by the US Supreme Court in which it was argued that the transaction would result in ‘greater and greater concentration of the beer industry into fewer and fewer hands’ [58]. The third blocked M&A case, in which a District Court granted the FTC’s request to block tobacco company Swedish Match’s acquisition of National Tobacco Company [59], took place in 2000. In this case, the FTC raised concerns about increasing market concentration, as well as how the transaction would potentially result in an increase in price for consumers [60].

In 13 M&A cases, we found that the transaction in question was approved subject to conditions. In many of these cases, one of the main reasons for imposing a remedy was to ostensibly address the ways in which the transaction would potentially harm consumers in the form of price increases and decreases in consumer choice. Three M&As involving beer company Anheuser-Busch InBev serve as illustrative examples. In these cases, US competition authorities contended that the transactions in question were problematic insofar as they would likely result in higher beer prices and fewer choices for consumers, including as a result of a reduction in ‘innovation’ for consumers [61–63]. In all three instances, various courts approved a set of remedies involving the divestiture of particular assets to address such harms [62, 64, 65].

The remaining 10 cases sourced from the two US competition authorities related to cartel behaviour. The primary concerns raised by the authorities in these cases centred on price-fixing typically in the direction of higher prices, and restrictions of trade. In all instances, the parties were ordered to restrain from taking any further part in the conduct in question.

### European Commission

We found 86 cases with documents published by the EC, dating back to 1999. Most of these cases (*n* = 72) related to M&As. The EC did not raise any competition-related concerns in 59 M&A cases, with these transactions approved without conditions.

The EC raised competition-related concerns in 13 M&A cases, with these transactions subsequently approved with conditions. In these cases, consumers were commonly portrayed as the group most likely to be harmed by such transactions, particularly in the form of price increases and a restriction in choice. As a pertinent example, in the proposed acquisition by Imperial Tobacco of Reemtsma, the EC expressed concern that ‘Imperial will be in a position to increase prices of own-label cigarettes, which will probably have the effect of increasing the prices of all brands’, or, alternatively, ‘to restrict supply volumes’ of own-label cigarette products, in the United Kingdom [66]. To remedy these concerns, the EC accepted the undertakings proposed by the parties in question involving, among other things, a commitment to continue supplying Reemtsma’s trademark to cigarette distributors upon request for a period of twenty years [66].

Consistent with the single market imperative behind EU competition policy [26], the EC often expressed concerns relating to restrictions of trade between Member States during its assessments of M&A cases. In such cases, competing firms, particularly those based in other Member States, were often considered to be at risk of being harmed. For instance, in its assessment of potential effects of a proposed merger between tobacco corporations Philip Morris International and Swedish Match, the EC contended that the transaction would potentially result in rival companies from being foreclosed in the supply of ‘factory made cigarettes’, ‘roll your own’ tobacco products, and heated tobacco products in Sweden [67]. In response, the authority accepted, inter alia, the merging parties’ proposed undertaking to divest Swedish Match’s distribution operations in the same country [67].

We also found five cases relating to price-fixing cartels. Again, in such cases the EC frequently perceived consumer harm in the form of price increases and, at a higher level, trade between Member States, as the main causes of concern. As an illustration, in its assessment of an alleged price-fixing cartel in the Belgian beer market, the EC reasoned that the main goal of the cartel was to ‘set prices at a higher level than would have happened in normal competition’, and that this was ‘to the detriment of consumers’, and was significant enough to have had an influence on the ‘pattern of trade between Member States’ [68]. In all cartel cases, the EC imposed fines on all or some of the parties involved.

One of the identified cases related to the abuse of a dominant position by beer corporation Anheuser-Busch InBev, which was found to have restricted imports of its own beer products from the Netherlands into Belgium [69]. In response, the EC issued a fine, and also accepted a commitment by the beer company to refrain from engaging in such conduct in the future [69].

## Discussion

This study analysed assessments and decisions made by competition authorities in four jurisdictions (Australia, South Africa, the US, the EU) with respect to cases involving three harmful consumer product industries (alcoholic beverages, soft drinks, tobacco). In cases where competition-related concerns were raised, we found that competition authorities in Australia, the US, and the EU commonly focused on alleged or potential harms to consumers in a neoclassical sense – that is, increased prices and restrictions in consumer choice – in their assessments and decision-making. The EC also raised concerns about the potential impact of the conduct in question on trade between Member States on several occasions. We found that South Africa’s competition authorities instead routinely considered potential harms to workers, so-called ‘Historically Disadvantaged Persons’ (with respect to share ownership), and domestic enterprises. This is in line with South Africa’s Competition Act, which mandates its competition authorities to assess various public interest effects of mergers. Across the four jurisdictions, we identified only three cases in which public health concerns were explicitly considered in the decision-making process, all of which involved the ACCC and related to agreements involving some form of coordination among competing companies.

### Implications for policy

The findings of this study have several important implications for policy. First, we identified that, at face value, many competition regulatory decisions informed by the ‘consumer welfare’ standard were inconsistent with ‘best practice’ public health policies targeting harmful consumer product industries [29, 70–73]. As a case in point, at a time when many European countries had at least some consumption-reduction measures in place to address alcohol-related harms [74], then-Commissioner of the European Commission justified the agency’s decision regarding the 2016 merger between Anheuser-Busch InBev and SABMiller on the grounds that even a ‘relatively small price increase [in beer] could cause considerable harm to consumers’ [75]. Clearly, consumer harm in the above comment is conceptualised in a considerably narrower way than how it is commonly conceptualised in the public health literature.

We argue, however, that the primary concern with the ‘consumer welfare’ standard from a public health perspective is not that it ostensibly promotes lower prices and increased output of harmful consumer products, as highlighted in the above example. Instead, the primary concern is that it tends to facilitate an increase in market power by delegitimising the consideration of a broad range of adverse consequences associated with market power (beyond the influence of market power on price and output). This is well illustrated, we argue, by the large number of merger approvals seemingly informed by the ‘consumer welfare’ standard identified in our study that involved no or minimal structural remediation. Clearly, the prices of harmful consumer products are important concerns and targets from a public health policy perspective. Nevertheless, excessive market power is fundamental to the growth and sustenance of these industries, and the failure of competition regulatory frameworks to address such power can be seen as a missed opportunity to tackle a major upstream cause of the ‘industrial epidemics’ [76] that these industries perpetuate [77].

Excessive market power in harmful consumer product industries drive and sustain ‘industrial epidemics’ in various ways. Such power, for instance, enables the firms in question to generate profits in excess of what can be generated in a competitive market environment [78]. Accordingly, the same firms have a greater capacity to accumulate and funnel resources towards shaping the market and regulatory environments in which they operate in their favour – that is, into an environment that promotes and compels the consumption of their products [4]. Excessive market power, which can be reinforced by and further reinforce financial and production economies of scale, can also translate into a structural and relational form of power in which the respective firms become increasingly important for governments to achieve their economic policy objectives [17, 79]. For some governments, this may create hesitancy to implement public health regulations out of fear of some form of retribution, such as capital and investment flight. It may also incentivise or compel some governments, particularly those of rich and powerful countries in the global North, to impede the policy space of other governments in order to protect the profit-making capacities of their corporations [80].

Notwithstanding the above points, the study has identified various precedents in which competition authorities have considered a wider range of social and political consequences of market power than what is prescribed by the ‘consumer welfare’ standard. These precedents arguably represent potential avenues through which competition regulation could work more synergistically with public health policies targeting harmful consumer product industries. As previously noted, we identified that Australia’s competition authority explicitly considered public health objectives in its decision-making in three coordination cases involving the tobacco and alcohol industries. These cases suggest that there is scope within Australia’s competition laws to consider public health. Integrating a similar approach into the regulation of mergers and abuses of market power could potentially yield a range of public health benefits.

Our findings also suggest that mandating the consideration of various public interest objectives in competition enforcement, as in the case of South Africa, may indirectly provide some public health benefits for particular social groups. More broadly, South Africa provides a useful blueprint on how to subordinate aspects of competition regulation to a social policy goal, such as redressing racial and socio-economic divisions perpetuated by exploitative systems (e.g., Apartheid) [31]. Moving forward, it could be worth calling for competition authorities in South Africa, along with competition authorities with similar mandates in other countries [81], to consider a wider range of harms when determining the extent to which the market power of firms active in harmful consumer product industries can undermine the welfare of disadvantaged social groups.

In the US, we identified a number of cases demonstrating the use of the country’s existing merger laws to challenge economic concentration in pursuit of multiple political and economic goals [82]. A notable example was the decision made by the US Supreme Court in 1966 to uphold a decision made by the DOJ to block a merger involving the 10th and 18th largest beer companies in the country at the time [58]. This decision provides a striking contrast to the subsequent decisions made by US competition authorities and courts to approve multiple large-scale mergers involving the world’s largest beer company, Anheuser-Busch InBev [62, 83, 84]. Promisingly, recent developments in the US, such as the publication of its latest merger guidelines in 2023 [85], suggest that the country’s merger laws may be in the process of being revived in accordance with how they were often applied in the post-Second World War period prior to the rise of the ‘consumer welfare’ standard [82]. Relatedly, it has been reported that several other jurisdictions, including Australia, Canada, and the United Kingdom, have begun various processes to strengthen their respective merger policies in a similar way [86–88].

Beyond updating national merger control frameworks, there is also a strong public health case to implement new mechanisms, such as a United Nations Competition Convention to govern cross-border mergers [89], to improve the regional and international coordination of merger control. Arguably, such mechanisms will likely be required to overcome the aggregate failure of merger control frameworks in diverse jurisdictions, especially in the global North, to address the consequences of excessive market power in harmful consumer product industries in the global South, wherein such power has often manifested as aggressive industry expansion and a corresponding surge in ill health and inequity [4, 79, 90–93].

### Strengths and limitations

A strength of this study was that it provided a novel and systematic approach to analysing a selection of assessments and decisions made by a sample of competition authorities through a public health lens. The study involved the analysis of a large number of documents, sourced from over the course of more than two decades, rarely analysed in public health research.

The study has a number of important limitations. First, to manage scope, we chose to focus on cases relating to four jurisdictions and three industries. Further insights would likely be provided by expanding this scope to cover a greater range of jurisdictions and industries of public health concern. Second, the analysis only focused on public enforcement cases because we felt this was an appropriate and important starting point, and also to manage scope. As such, the analysis did not include private enforcement cases, an area which warrants attention in future work. Third, our ability to systematically identify cases was limited by the design and functionality of the search and filter tools on the websites of the selected competition authorities, as well as the date range of the records archived. Nevertheless, we felt that the tools enabled us to find a comprehensive selection of relevant cases. Fourth, we did not examine the impacts of any of the competition regulatory decisions made, again mostly to manage scope. In this respect, a potential avenue for future research could be to analyse a range of social, economic, political, and technological impacts of competition regulatory decisions pertaining to major cases in one or more industries of public health concern. Fifth, we only analysed documents published in English. The overwhelming majority of documents published by the EC appeared to be published in English, which could be partly explained by the fact that the EC mostly focuses on competition cases involving at least two Member States, and because English is one of the official working languages within the EC. Nevertheless, we acknowledge that exclusively focusing on documents in English may have resulted in some cases being excluded from the analysis.

## Conclusion

In recent decades, competition authorities in Australia, the US, and the EU have made many decisions ostensibly oriented towards increasing the affordability and accessibility of alcohol beverages, soft drinks, and tobacco products. At face value, this is an objective that appears to be inconsistent with the objective of various public health policies seeking to minimise consumption-related harms associated with the same products in question. Nevertheless, it could be argued that, from a public health perspective, the main competition regulatory decisions of concern are not necessarily those oriented towards such a conflicting objective, but instead are those that passively facilitate the concentration of market power both within and across jurisdictional boundaries. Accordingly, we argue that increasing coherence between competition regulation and ‘best practice’ public health policies targeting harmful consumer product industries will likely need to involve providing competition authorities and courts with the necessary mandate and powers to regulate market power in the pursuit of multiple goals, potentially including, but not limited to, protecting and promoting the public’s health.

## Data Availability

The datasets used and/or analysed during the current study are available from the corresponding author on reasonable request.

## Abbreviations

ACCC: Australian Competition and Consumer Commission
CTSA: Competition Tribunal of South Africa
CUB: Carlton and United Breweries
DOJ: Department of Justice
EC: European Commission
EU: European Union
FTC: Federal Trade Commission
M&As: Mergers and acquisitions
US: United States
USD: United States dollars

## References

[1] Gilmore AB, Fabbri A, Baum F, Bertscher A, Bondy K, Chang HJ, et al. Defining and conceptualising the commercial determinants of health. Lancet. 2023. 10.1016/S0140-6736(23)00013-2. 10.1016/S0140-6736(23)00013-2 36966782

[2] Knai C, Petticrew M, Capewell S, Cassidy R, Collin J, Cummins S, et al. The case for developing a cohesive systems approach to research across unhealthy commodity industries. BMJ Glob Health. 2021;6.10.1136/bmjgh-2020-003543 10.1136/bmjgh-2020-003543 PMC7888371 33593757

[3] Global Burden of Disease Collaborative Network. Global burden of disease study results: Institute for Health Metrics and Evaluation, University of Washington; 2019. Available from: https://ghdx.healthdata.org/gbd-2019. 21 Nov 2023

[4] Moodie R, Stuckler D, Monteiro C, Sheron N, Neal B, Thamarangsi T, et al. Profits and pandemics: prevention of harmful effects of tobacco, alcohol, and ultra-processed food and drink industries. Lancet. 2013;381:670–9. 10.1016/s0140-6736(12)62089-3. 23410611 10.1016/S0140-6736(12)62089-3

[5] Jahiel RI. Corporation-induced diseases, upstream epidemiologic surveillance, and urban health. J Urban Health. 2008;85:517–31. 10.1007/s11524-008-9283-x. 18437580 10.1007/s11524-008-9283-x PMC2443251

[6] Stuckler D, McKee M, Ebrahim S, Basu S. Manufacturing epidemics: the role of global producers in increased consumption of unhealthy commodities including processed foods, alcohol, and tobacco. PLoS Med. 2012;9: e1001235. 10.1371/journal.pmed.1001235. 22745605 10.1371/journal.pmed.1001235 PMC3383750

[7] Lee K, Freudenberg N. Public Health Roles in Addressing Commercial Determinants of Health. Annu Rev Public Health. 2022. 10.1146/annurev-publhealth-052220-020447. 34982584 10.1146/annurev-publhealth-052220-020447

[8] Peruga A, Lopez MJ, Martinez C, Fernandez E. Tobacco control policies in the 21st century: achievements and open challenges. Mol Oncol. 2021;15:744–52. 10.1002/1878-0261.12918. 33533185 10.1002/1878-0261.12918 PMC7931122

[9] Wyper GMA, Mackay DF, Fraser C, Lewsey J, Robinson M, Beeston C, et al. Evaluating the impact of alcohol minimum unit pricing on deaths and hospitalisations in Scotland: a controlled interrupted time series study. Lancet. 2023;401:1361–70. 10.1016/S0140-6736(23)00497-X. 36963415 10.1016/S0140-6736(23)00497-X PMC10154457

[10] Andreyeva T, Marple K, Marinello S, Moore TE, Powell LM. Outcomes following taxation of sugar-sweetened beverages: a systematic review and meta-analysis. JAMA Netw Open. 2022;5:e2215276. 10.1001/jamanetworkopen.2022.15276. 35648398 10.1001/jamanetworkopen.2022.15276 PMC9161017

[11] Friel S, Collin J, Daube M, Depoux A, Freudenberg N, Gilmore AB, et al. Commercial determinants of health: future directions. Lancet. 2023. 10.1016/S0140-6736(23)00011-9. 10.1016/S0140-6736(23)00011-9 36966784

[12] Buse K, Tomson G, Kuruvilla S, Mahmood J, Alden A, van der Meulen M, et al. Tackling the politics of intersectoral action for the health of people and planet. BMJ. 2022. 10.1136/bmj-2021-068124. 10.1136/bmj-2021-068124 PMC8790677 37462013

[13] Paul S. Antitrust as allocator of coordination rights. UCLA Law Rev. 2020;67. Available at SSRN: https://ssrn.com/abstract=3337861

[14] Meagher M. Adaptive Antitrust. ABA Spring Meeting 2021, Course Materials 2021.

[15] Meagher M. Competition is killing us: how big business is harming our society and planet - and what to do about it. London: Penguin Group; 2020.

[16] Teachout Z, Khan L. Market structure and political law: a taxonomy of power. Duke J Const Law Public Policy. 2017;9:37–74.

[17] Fuchs D, Lederer M. The power of business. Bus Politics. 2007;9:1–17.

[18] Wood B, Baker P, Sacks G. Conceptualising the commercial determinants of health using a power lens: a review and synthesis of existing frameworks. Int J Health Policy Manage. 11. 10.34172/ijhpm.2021.05. 10.34172/ijhpm.2021.05 PMC9808328 33619932

[19] Cowgill B, Prat A, Valletti T. Political Power and Market Power. CEPR Discussion Paper No. DP17178. 2023.

[20] Callander S, Foarta D, Sugaya T. Market Competition and Political Influence: an Integrated Approach. Econometrica. 2022;90:2723–53. 10.3982/ecta19775.

[21] Crouch C. The strange non-death of neoliberalism. Cambridge, UK: Polity; 2011.

[22] Stiglitz J. Towards a broader view of competition policy. In: Bonakele T, Fox E, Mncube L, editors. Competition policy for the new era: insights from the BRICS countries. Oxford: Oxford Publishing; 2017. pp. 4–21.

[23] Khan L. The ideological roots of America’s market power problem. Yale Law J Forum. 2018;127:960.

[24] Lancieri F, Posner E, Zingales L. The political economy of the decline of antitrust enforcement in the United States. Cambridge, MA: National Bureau of Economic Research; 2022.

[25] Newman JM. The output-welfare fallacy: a modern antitrust paradox. Iowa Law Rev. 2022;107:563–619.

[26] Buch-Hansen H, Wigger A. The politics of European competition regulation: a critical political economy perspective. London and New York: Routledge; 2011.

[27] OECD. Consumer welfare standard - advantages and disadvantages compared to alternative standards. 2023.

[28] Crane DA. Harmful output in the antitrust domain: lessons from the tobacco industry. Georgia Law Rev. 2005;39:321.

[29] World Health Organization. The SAFER technical package: five areas of intervention at national and subnational levels. Geneva: WHO; 2019.

[30] Freyer T. Antitrust and global capitalism, 1930–2004. Cambridge, UK: Cambridge Universtiy; 2006.

[31] Njisane Y, Ratshisusu H. Public interest issues in cross-border mergers. In: Bonakele T, Fox E, Mncube L, editors. Competition policy for the new era: insights from the BRICS countries. Oxford: Oxford University Press; 2017.

[32] Ezrachi A, Zac A, Decker C. The effects of competition law on inequality—an incidental by-product or a path for societal change? J Antitrust Enforc. 2023;11:51–73. 10.1093/jaenfo/jnac011.

[33] Uwadi E. A case for public interest considerations in merger control analysis with reference to competition law enforcement in developing countries: the example of South Africa. Transnational Dispute Management; 2023 [29 August 2023]. Available from: https://www.transnational-dispute-management.com/article.asp?key=3003.

[34] OECD. Market Definition. Paris: Organisation for Economic Co-operation and Development. 2012. Contract No.: DAF/COMP(2012)13/REV1.

[35] Australian Competition and Consumer Commission. Public Registers. Available from: https://www.accc.gov.au/public-registers. 27 Oct 2023.

[36] Competition Tribunal of South Africa. Decided Cases. Available from: https://www.comptrib.co.za/cases-archived. 27 Oct 2023.

[37] Antitrust Division. Antitrust case filings: U.S. Department of Justice. 2023. https://www.justice.gov/atr/antitrust-case-filings. 27 Oct 2023.

[38] Federal Trade Commission. Legal library: cases and proceedings: United States Government; 2023. Available from: https://www.ftc.gov/legal-library/browse/cases-proceedings. 27 Oct 2023.

[39] European Commission. Competition: Case documents. Available from: https://ec.europa.eu/competition/publications/cases_en.html. 27 Oct 2023.

[40] ACCC. Statement of issues: Woolworths Limited - proposed acquisition of Lindisfarne Cellars. Canberra: Australian Competition and Consumer Commission; 2013.

[41] ACCC. Statement of issues: Woolworths Limited - proposed acquisition of a takeaway packaged liquor licence located at Rocherlea. Canberra: Australian Competition and Consumer Commission; 2012.

[42] ACCC. Acquirer: Coca Cola Amatil Ltd; Target: Berri Ltd. Canberra: Australian Competition & Consumer Commission; 2003. https://www.accc.gov.au/public-registers/mergers-registers/public-informal-merger-reviews/acquirer-coca-cola-amatil-ltd-target-berri-ltd. 12 Apr 2024.

[43] ACCC. Statement of issues: Woolworths Limited - proposed acquisition of the Karabar Supabarn supermarket. Canberra: Australian Competition & Consumer Commission; 2008.

[44] ACCC. Public Competition Assessment. Asahi Group Holdings – proposed acquisition of Carlton & United Breweries. Canberra: Australian Competition & Consumer Commission; 2020.

[45] ACCC. Public Competition Assessment. Scandinavian Tobacco Group A/S - proposed acquisition of Swedish Match AB. Canberra: Australian Competition & Consumer Commission; 2010.

[46] ACCC. Form G: Notification of exclusive dealing. Canberra: Australian Competition & Consumer Commission; 2015.

[47] ACCC. Determination: application for authorisation lodged by British American Tobacco Australia Limited, Imperial Tobacco Australia Limited and Philip Morris Limited. Canberra: Australian Competition & Consumer Commission; 2017.

[48] ACCC. Applications for authorisation lodged by the state of Queensland acting through the Office of Liquor and Gaming Regulation. Canberra: Australian Competition & Consumer Commission; 2010.

[49] ACCC. Application for revocation and substitution of authorisations A91224 & A91225 lodged by the state of Queensland acting through the Office of Liquor and Gaming Regulation. Canberra: Australian Competition & Consumer Commission; 2014.

[50] Competition Tribunal of South Africa. Case No: 35/LM/Apr11. Pretoria: Competition Tribunal of South Africa; 2011.

[51] Competition Tribunal of South Africa. Case No: 08/LM/Feb02. Pretoria: Competition Tribunal of South Africa; 2002.

[52] Competition Tribunal of South Africa. Case No: LM262Jan18. Pretoria: Competition Tribunal of South Africa; 2018.

[53] Competition Tribunal of South Africa. CT case no: LM187Oct19. Pretoria: Competition Tribunal of South Africa; 2019.

[54] Competition Tribunal of South Africa. Case No: LM243Mar15. Pretoria: Competition Tribunal of South Africa; 2015.

[55] Competition Tribunal of South Africa. Case No: LM211Jan16. Pretoria: Competition Tribunal of South Africa; 2016.

[56] United States of America v. R.J. Reynolds Tobacco Company et al. Civil Action No.1688-70 (United States District Court for the District of New Jersey). 1970.

[57] United States of America v. Pabst Brewing Company et al. Civil Acton No. 59 C 215 (United States District Court for the Eastern District of Wisconsin). 1971.

[58] United States of America v. Pabst Brewing Co. 384 U.S. 546 (United States Supreme Court). 1966.

[59] FTC v. Swedish Match. 131 F. Supp 2d 151 (United States District Court for the District of Columbia). 2000.

[60] FTC. Administrative complaint: Swedish Match North America Inc., and National Tobacco Company, L.P. Washington, DC: Federal Trade Commission; 2000.

[61] United States Department of Justice Antitrust Division. Competitive Impact Assessment: U.S v. Anheuser-Busch InBev SA/NV and SABMiller PLC. Washington, DC: U.S. Department of Justice; 2016.

[62] United States of America v. Anheuser-Busch InBev SA/NV., et al. Civil Action No. 13-127 (United States District Court for the District of Columbia). 2013.

[63] United States Department of Justice Antitrust Division. Competitive Impact Assessment: U.S. v. Anheuser-Busch InBev SA/NV, et al. Washington, DC: U.S. Department of Justice; 2020.

[64] United States of America v. Anheuser-Busch InBev SA/NV and SABMiller PLC. Civil Action No. 16-1483 (United States District Court for the District of Columbia). 2018.

[65] United States of America v. Anheuser-Busch InBev SA/NV, et al. Civil Action No. 4:20-cv-01282-SRC (United States District Court for the Eastern District of Missouri Eastern Division). 2021.

[66] European Commission. Case COMP/M.2779 - Imperial tobacco / Reemtsma cigarettenfabriken. Brussels: European Commission; 2002.

[67] European Commission. Case M.10792 - Philip Morris International / Swedish Match. Brussels: European Commission; 2022.

[68] European Commission. Case IV/37.614/F3 PO/Interbrew and Alken-Maes. Brussels: European Commission; 2001.

[69] European Commission. Case AT.40134. Brussels: European Commission; 2019.

[70] WHO. WHO manual on sugar-sweetened beverage taxation policies to promote healthy diets. Geneva: World Health Organization; 2022.

[71] WHO Framework Convention on Tobacco Control. Guidelines for implementation of Article 13 of the WHO Framework Convention on Tobacco Control (Tobacco advertising, promotion and sponsorship). Geneva: World Health Organization; 2008. Available from: https://www.who.int/fctc/guidelines/article_13.pdf. 27 May 2020.

[72] WHO. WHO report on the global tobacco epidemic, 2023: protect people from tobacco smoke. Geneva: World Health Organization; 2023.

[73] WHO. Global alcohol action plan 2022–2030. Geneva: World Health Organization; 2023.

[74] Eurocare. European report on Alcohol Policy: a review. Brussels: European Alcohol Policy Alliance; 2016.

[75] European Commission. Daily News 25 / 05 / 2016 2016. Available from: https://ec.europa.eu/commission/presscorner/detail/en/MEX_16_1909. 24 Nov 2023.

[76] Majnoni d’Intignano B. Épidémies industrielles. Commentaire. 1995;71:557–65.

[77] Wood B, Williams O, Baker P, Nagarajan V, Sacks G. The influence of corporate market power on health: exploring the structure-conduct-performance model from a public health perspective. Global Health. 2021;17.10.1186/s12992-021-00688-2 10.1186/s12992-021-00688-2 PMC8025506 33823900

[78] Wood B, Williams O, Nagarajan V, Sacks G. Market strategies used by processed food manufacturers to increase and consolidate their power: a systematic review and document analysis. Globalization Health. 2021;17.10.1186/s12992-021-00667-7. 10.1186/s12992-021-00667-7 PMC7836045 33499883

[79] Baker P, Machado P, Santos T, Sievert K, Backholer K, Hadjikakou M, et al. Ultra-processed foods and the nutrition transition: global, regional and national trends, food systems transformations and political economy drivers. Obes Rev. 2020. 10.1111/obr.13126. 32761763 10.1111/obr.13126

[80] Milsom P, Smith R, Baker P, Walls H. Corporate power and the international trade regime preventing progressive policy action on non-communicable diseases: a realist review. Health Policy Plan. 2020. 10.1093/heapol/czaa148. 10.1093/heapol/czaa148 PMC8128013 33276385

[81] Naidu L, Tzarevski A, Nxumalo S. Africa: The increasing focus on public interest concerns in competition policy: Global Compliance News; 2023. Available from: https://www.globalcompliancenews.com/2023/06/02/https-insightplus-bakermckenzie-com-bm-antitrust-competition_1-africa-the-increasing-focus-on-public-interest-concerns-in-competition-policy_05302023/. 8 Dec 2023.

[82] Fox E. Eleanor Fox: a slice of forgotten history and its light on the future – changing the lens on antitrust: Promarket; 2023. Available from: https://www.promarket.org/2023/08/14/eleanor-fox-a-slice-of-forgotten-history-and-its-light-on-the-future-changing-the-lens-on-antitrust/. 14 Dec 2023.

[83] United States of America v. Anheuser-Busch InBev SA/NV, and SABMiller PLC. Civil Action No. 1:16-cv-01483 (United States District Court for the District of Columbia). 2016.

[84] United States of America v. InBev NV/SA, et al. Civil Action No. 1:08-cv-01965 (United States District Court for the District of Columbia). 2008.

[85] U.S. Department of Justice and the Federal Trade Commission. Merger Guidlines. Washington, DC: 2023.

[86] UK Government. Reforming competition and consumer policy: government response: Department for Business, Energy & Industrial Strategy; 2022. Available from: https://www.gov.uk/government/consultations/reforming-competition-and-consumer-policy/outcome/reforming-competition-and-consumer-policy-government-response. 19 Sept 2022.

[87] ACCC. Reform of merger laws critical for Australia’s economic transition: Australian Competition & Consumer Commission; 2023. Available from: https://www.accc.gov.au/media-release/reform-of-merger-laws-critical-for-australia%E2%80%99s-economic-transition. 15 May 2023.

[88] Competition Bureau of Canada. Important amendments to the Competition Act come into effect: Government of Canada; 2022. Available from: https://www.canada.ca/en/competition-bureau/news/2022/06/important-amendments-to-the-competition-act-come-into-effect.html. 14 Dec 2023.

[89] ETC Group. Agribusiness mega-mergers expose need for UN Competition Convention. Montréal: ETC Group; 2017.

[90] Jernigan DH, Babor TF. The concentration of the global alcohol industry and its penetration in the African region. Addiction. 2015;110:551–60. 10.1111/add.12468. 25771689 10.1111/add.12468

[91] Lee K, Eckhardt J. The globalisation strategies of five Asian tobacco companies: an analytical framework. Glob Public Health. 2017;12:269–80. 10.1080/17441692.2016.1251604. 27884083 10.1080/17441692.2016.1251604 PMC5553427

[92] Collin J, Hill SE, Smith KE. Merging alcohol giants threaten global health. BMJ. 2015;351:h6087. 10.1136/bmj.h6087. 26578535 10.1136/bmj.h6087

[93] Hanefeld J, Hawkins B, Knai C, Hofman K, Petticrew M. What the InBev merger means for health in Africa. BMJ Glob Health. 2016;1:e000099. 10.1136/bmjgh-2016-000099. 28588945 10.1136/bmjgh-2016-000099 PMC5321335

